# The possible roles of B‐cell novel protein‐1 (BCNP1) in cellular signalling pathways and in cancer

**DOI:** 10.1111/jcmm.12989

**Published:** 2016-09-29

**Authors:** Sapan J. Patel, Gaurang L. Trivedi, Costel C. Darie, Bayard D. Clarkson

**Affiliations:** ^1^ Memorial Sloan Kettering Cancer CenterMolecular Pharmacology and Chemistry ProgramNew YorkNYUSA; ^2^ Department of Chemistry and Biomolecular ScienceClarkson UniversityBiochemistry and Proteomics GroupPotsdamNYUSA; ^3^ Cold Spring Harbor LaboratoryCold Spring HarborNYUSA

**Keywords:** phoshoinositide 3‐kinase, B‐cell novel protein‐1, family member of 129C, bioinformatics, p38 MAPK

## Abstract

B‐cell novel protein‐1 (BCNP1) or Family member of 129C (FAM129C) was identified as a B‐cell‐specific plasma‐membrane protein. Bioinformatics analysis predicted that BCNP1 might be heavily phosphorylated. The BCNP1 protein contains a pleckstrin homology (PH) domain, two proline‐rich (PR) regions and a Leucine Zipper (LZ) domain suggesting that it may be involved in protein‐protein interactions. Using The Cancer Genome Atlas (TCGA) data sets, we investigated the correlation of alteration of the BCNP1 copy‐number changes and mutations in several cancer types. We also investigated the function of BCNP1 in cellular signalling pathways. We found that BCNP1 is highly altered in some types of cancers and that BCNP1 copy‐number changes and mutations co‐occur with other molecular alteration events for TP53 (tumour protein P53), PIK3CA (Phosphatidylinositol‐4,5‐Bisphosphate 3‐Kinase, Catalytic Subunit Alpha), MAPK1 (mitogen‐activated protein kinase‐1; ERK: extracellular signal regulated kinase), KRAS (Kirsten rat sarcoma viral oncogene homolog) and AKT2 (V‐Akt Murine Thymoma Viral Oncogene Homolog 2). We also found that PI3K (Phoshoinositide 3‐kinase) inhibition and p38 MAPK (p38 mitogen‐activated protein kinase) activation leads to reduction in phosphorylation of BCNP1 at serine residues, suggesting that BCNP1 phosphorylation is PI3K and p38MAPK dependent and that it might be involved in cancer. Its degradation depends on a proteasome‐mediated pathway.

## Introduction

Plasma membrane proteins are potential therapeutic targets in various diseases like cancer 1. One example of such proteins is BCNP1 (also termed FAM129C). BCNP1 is a member of a small family of poorly understood proteins that might be involved in B‐cell development, function, and other malignancies. Other members of the family include Niban (FAM129A) 2 and FAM129B (MINERVA “Melanoma IN‐VAsion by ERK”) 3. BCNP1 was initially discovered by Boyd and colleagues in their study of the B‐cell surface plasma membrane proteins that might be potential targets for leukaemia therapy 1. In this study, they cloned the entire BCNP1 gene from normal spleen and cDNA library of Daudi cell line and also found that BCNP1 was highly expressed in multiple leukaemia and lymphoma patient samples and cell lines compared to normal tissues. BCNP1 expression was found to be restricted to B‐cell rich normal tissues and was particularly high in lymph nodes, spleen and thymus 1. BCNP1 was also identified in the B‐cells in the large scale study by Kim *et al*. involving an enormous effort to catalogue the human proteome performed with high‐resolution Fourier‐transform mass spectrometry 4. It has been more than a decade since BCNP1 was identified; yet the role of BCNP1 in B‐cell development and malignancies is still unknown. The present study focused on role of BCNP1 in cancer and cell signalling, particularly PI3K and p38 MAPK pathways.

PI3Ks are involved in multiple regulatory processes such as cell survival, proliferation and differentiation. Many components of the PI3K signalling pathways are frequently altered in a broad spectrum of human cancers 5. Currently, there are multiple clinical trials studying the role of inhibitors involving PI3K and other kinases in the signalling pathways in cancer 5, 6.

The structure and normal function of BCNP1 is not known. In addition, the relationship between the BCNP1 and cancer is also not very well understood. However, it is well‐known that some members of the FAM129 proteins (to which BCNP1 or FAM129C belongs) such as Niban (FAM129A) are indeed involved in cancer. For example, Niban was found to be up‐regulated in renal carcinoma 2, 7, 8 in renal interstitial fibrosis 6, head and neck squamous cell carcinoma and squamous dysplasia 9, and also in thyroid tumours and Hashimoto's thyroiditis 10. Sun *et al*. found that Niban is involved in the endoplasmic reticulum stress response by decreasing phosphorylation of p70 ribosomal S6 subunit kinase (S6K) 1 and eukaryotic initiation factor 4E‐binding protein1 (4E‐BP1) 11. They also found that Niban‐specific siRNA suppression promoted apoptosis in HeLa cells by affecting translation 11. Niban inhibits apoptosis by a p53‐dependent mechanism that destabilizes p53 upon genotoxic stress such as ultraviolet (UV) radiation 12.

The proline rich region of FAM129B at the carboxyl end is phosphorylated at six serine residues 3. Endogenous knockdown of FAM129B also reduces invasion in a three‐dimensional collagen matrix based assay system in melanoma cells 3. Old and colleagues also showed that ERK/MAPK dependent phosphorylation of FAM129B is responsible for promoting tumour cell invasion into collagen matrices 3. FAM129B is also involved in TNFα‐dependent and anti‐CD95‐dependent apoptosis in HeLa cells 13 and in Wnt/β‐catenin signalling pathway‐dependent apoptosis in melanoma cells 14. FAM129B mutant mice showed delayed wound healing because of alteration of wound healing related genes necessary for cell motility 15.

To understand the collective behaviour of cancer cells, we are studying the Ph+ Acute Lymphoblastic Leukaemia cell line (ALL3) that has excellent growth at high starting cell densities (HD) and no growth at low starting cell densities (LD) 16, 17. In our study, we found that LD ALL3 cells are unresponsive to any known cytokines or combinations and only grow in culture when stimulated by diffusible factors (cell‐free filtered supernatant) secreted by ALL3 cells growing at HD (HDSN). In our microarray study, we found that BCNP1 was the most highly up‐regulated gene (~27, 49, and 121‐fold, respectively, on days 1, 3 and 6) upon stimulation of the LD ALL3 cells by diffusible factors in HD ALL3 HDSN 16, 17. A recent study by Yuki *et al*. showed that overexpression of a zinc‐finger protein (ZNF777) inhibited proliferation of cells at low density through down‐regulation of FAM129A (Niban) 18. This finding and our finding that there is striking up‐regulated expression of BCNP1 on stimulation of LD ALL3 cells by HDSN of HD ALL3 cells strongly suggests that BCNP1 might have an important role in cell density‐dependent cell proliferation at least in B‐cells.

In the current study, we employed bioinformatics and biochemical approaches to investigate the functions of BCNP1, its relationship with various types of cancer and its biochemical relationship with PI3K and p38 MAPK pathways. Bioinformatics analysis predicted that BCNP1 contains a pleckstrin homology (PH) domain, two proline‐rich (PR) regions, and Leucine Zipper domain (LZ) suggesting that it may be involved in protein‐protein interactions. Additional bioinformatics analysis also suggested that BCNP1 is phosphorylated. When we investigated the correlation of alteration of the BCNP1 copy‐number changes and mutations in several cancer types using TCGA data sets, we concluded that BCNP1 is highly altered in some of the cancer types and that BCNP1 copy‐number changes and mutations co‐occur with other molecular alteration events for TP53, PIK3CA, MAPK1, KRAS and AKT2. In addition, when we investigated the function of BCNP1 in the PI3K‐dependent signalling pathway, we found that PI3K inhibition and p38 MAPK activation leads to reduction in serine‐phosphorylation of BCNP1 suggesting that BCNP1 phosphorylation is dependent on PI3K and p38 MAPK and it might be involved in cancer. The biomedical significance of our findings is discussed.

## Material and methods

### Cell line

HEK293 cells were cultured in Dulbecco's modified eagle medium (DMEM) supplemented with 10% heat‐inactivated foetal bovine serum (FBS) and penicillin and streptomycin. Cells were kept at 37°C, 5% CO_2_ in incubator at desired times depending on type of experiments. HEK293 cells were used in this study because they are amenable system for transfection.

### Antibodies and reagents

Anti‐FLAG M2 monoclonal antibody (Sigma F1804) and Anti‐phosphoserine (Sigma P3430) antibody were purchased from Sigma‐Aldrich, St. Louis, MO, USA. Anti‐phospho Akt (Ser473) (CST#9271) and anti‐Akt (CST#9272) were from Cell Signaling Technology, Boston, MA, USA. Anti‐phospho p38 MAPK (Thr180/Tyr182) and anti‐MAPK were from Cell Signaling Technology, USA. All secondary antibodies and anti‐βactin antibody were from Abcam, Cambridge, MA, USA. Polyethyleneimine (PEI) was purchased from Polyscience Inc, Warrington, PA, USA. Protein A/G Plus Agarose beads (sc2003) for immunoprecipitation (IP) were from Santa Cruz Biotechnology, USA. The compounds wortmannin and LY294002 were from Selleck chemicals, Houston, TX, USA. Cycloheximide, sorbitol and MG132 were from Sigma‐Aldrich.

### BCNP1 (FAM129C) cDNA construct

The BCNP1 (FAM129C) (NM_173544.4) cDNA construct (HsCD00295103) cloned into pENTR223.1 was obtained from Harvard Medical School Plasmid DNA core facility, Boston, MA, USA. The pcDNA3 Flag HA empty vector [from William Sellers (Addgene plasmid # 10792)] was obtained from Addgene, Cambridge, MA, USA. The FAM129C cDNA was amplified by PCR. The PCR product contained EcoRI and NotI restriction sites at 5′‐ and 3′‐end, respectively. The Empty vector and PCR product were digested using EcoRI and NotI (New England Biolabs, Ipswich, MA, USA: NEB) enzymes, ran on the 1% agarose gel and purified using gel extraction kit (#28704) from Qiagen, Valencia, CA, USA. After purification, the PCR product was ligated into pcDNA3 Flag HA and transformed into 5‐α highly competent E. Coli as described in a protocol from NEB. A few colonies were selected to confirm the presence of the desired cDNA construct. The sequence fidelity was confirmed using Sanger‐sequencing method at DNA Sequencing Core Facility at Memorial Sloan‐Kettering Cancer Center, NY‐USA (MSKCC).

### Transfection

Transient transfection of pcDNA3 Flag HA‐FAM129C was carried out using PEI. The HEK293 cells were plated 24 hrs prior to transfection. For transfection into 60 mm plate 3 μg of plasmid DNA was diluted in 100 μl high glucose serum‐free Dulbecco's modified eagle medium (DMEM). To dilute PEI, 0.75 μl of 1 mg/ml stock solution was added in 100 μl high glucose serum‐free DMEM. Diluted PEI solution was added to diluted DNA and incubated at room temperature (RT) for 30 min. After 30 min., DNA/PEI mixture was added drop‐wise to HEK293 cells and incubated at 37°C for 24–48 hrs in tissue culture incubator and processed further.

### Immunoprecipitation and western blotting

Transiently transfected cells were washed twice with ice cold phosphate buffered saline (PBS) and scraped with 1–2 ml of ice cold PBS and centrifuged at ~525 g for 8–10 min. at 4°C using eppendorf 5810 high speed centrifuge. The pelleted cells were lysed with 0.2–0.4 ml of RIPA lysis buffer [radioimmunoprecipitation assay buffer (Santa Cruz Biotech, Dallas, TX, USA #SC‐24948) with protease and phosphatase inhibitors cocktails for 30 min. on ice and centrifuged at 4°C for 10 min. at 13,500 g eppendorf 5810 high speed centrifuge. The lysates containing supernatants were collected carefully without disturbing the pellets. Protein concentration of the supernatant was measured using a Pierce Protein Assay Kit (Life Technologies, Carlsbad, CA, USA # 23225). For IP, 500 μg of proteins were incubated with anti‐flag antibody at 4°C overnight. The protein–antibody immunoprecipitates were collected by protein A/G plus‐agarose (Santa Cruz Biotech, Texas, USA #SC‐2003). Following the final wash, the samples were boiled and centrifuged to pellet the agarose beads. To check protein levels from whole cell lysates (WCL), 50 μg of proteins were used for analysing for western blotting. All the protein samples were prepared in 2× Laemmali sample buffer (Biorad#1610737). WCL and immunoprecipitated (IP) proteins were separated by Sodium Dodecyl Sulphate Poly‐acrylamide Gel Electrophoresis (SDS‐PAGE) at RT and transferred to polyvinylidene fluoride (PVDF) membrane using BioRad transfer apparatus at 4°C. The membranes were blocked with 3% bovine serum albumin (BSA) or 5% non‐fat milk in TBS (tris‐buffered saline) with 0.1% Tween‐20 (TBST) for 60–90 min. Membranes were probed with a variety of primary antibodies at 4°C overnight. The membranes were washed three times with TBST, and developed with horseradish peroxidase‐conjugated (HRP) anti‐mouse or anti‐rabbit antibody (Abcam, USA) (1:10,000 dilution) for 1 hr at RT. Membranes were then washed three times with TBST and developed with a Western Lightning Chemiluminescence reagent (ECL, Perkin Elmer, Waltham, MA, USA) for 1–5 min. at RT, and the signals were developed on film. The films were scanned and intensity was measured and bands were transformed using Image J (1.8.0_77; 64 bit) software available from National Institute of Health, USA (NIH).

### TCGA expression analysis of BCNP1

We used comprehensive data available for cancer samples as part of the TCGA (The Cancer Genome Atlas) project (tcga.cancer.gov). All TCGA data were from the cBio Cancer Genomics Portal (www.cbioportal.org); the data were collected on July 2015. We used OncoPrint tool to visualize the data 19, 20.

### Half‐life determination

Unmanipulated or transfected cells were exposed to 20 mg/ml cycloheximide and harvested at different time points, and the extracts were analysed by WB.

## Results & discussion

### BCNP1 is a novel protein

**Figure 1:**
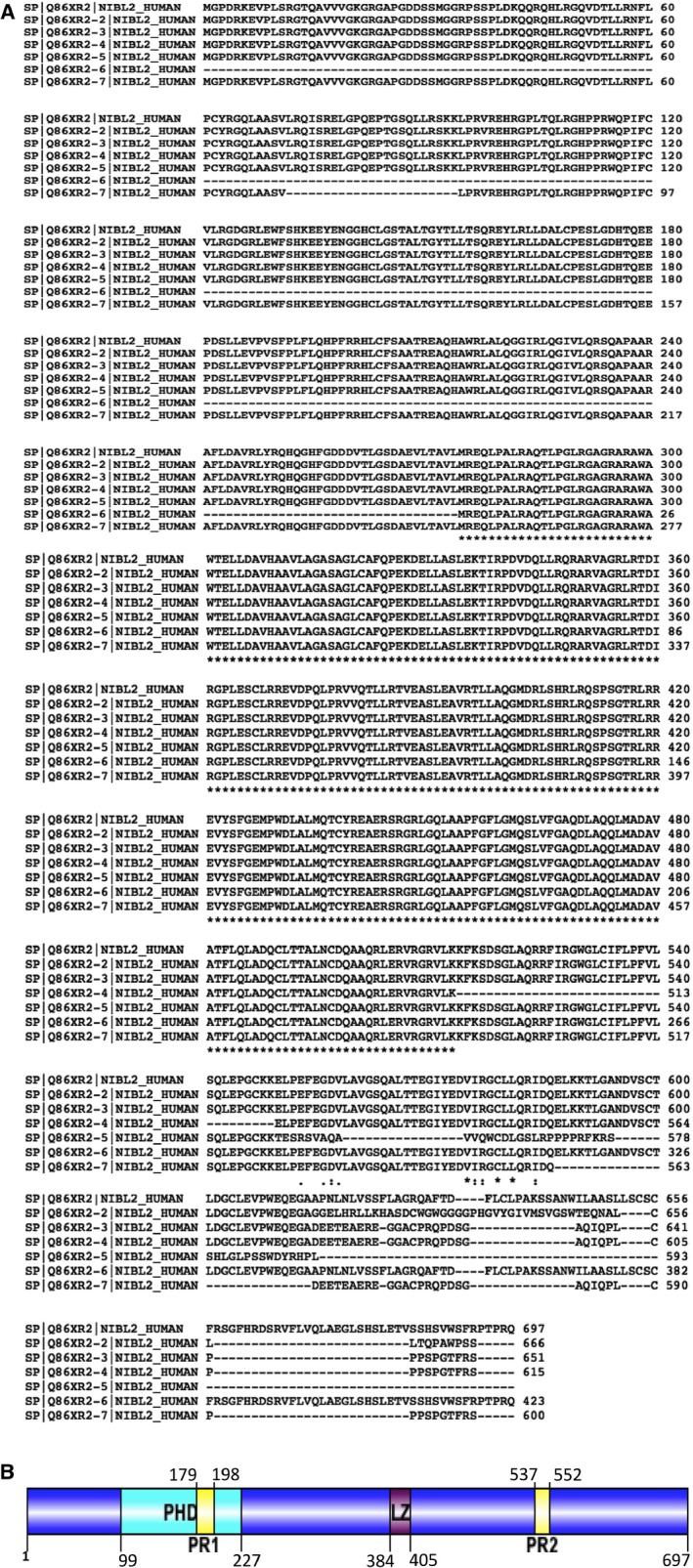
**(A)** Multiple alignments of different BCNP1 isoforms using CLUSTALW2. **(B)** Schematic representation of domain organization in BCNP1. BCNP1 modular organization is represented along with its major domains: PH domains: pleckstrin homology domain; PR1: proline rich region 1; PR2: proline rich region 2; LZ: Leucine Zipper domain.

BCNP1 is located on chromosome 19p13.11 position with 21 exons. There are seven BCNP1 isoforms each with different spliced exon encoding proteins of 697, 666, 651, 615, 593, 423, and 600 amino acids (UniPort accession# Q86XR2‐1, Q86XR2‐2, Q86XR2‐3, Q86XR2‐4, Q86XR2‐5, Q86XR2‐6 and Q86XR2‐7, respectively) as shown in Figure 1A. The BCNP1 gene is conserved in chimpanzee, cow, mouse, dog and rat.

BCNP1 contains a PH domain (Fig. 1B). The PH domain was originally identified in pleckstrin, the major substrate of protein kinase C in platelets in 1993 21. The PH domain consists of approximately ~130 residues and is found in a variety of organisms from yeast to humans. PH domains are found in about 252 human proteins and it is the 11th most abundant domain in the human genome 22. PH domains are best known for their ability to bind phosphoinositides and to target their host proteins to cellular membranes 23. Their functions appear to be very diverse, including cellular signalling, cytoskeleton organization, membrane trafficking and phospholipid modifications 24.

BCNP1 also has two PR regions located on N and C‐terminals of the protein (Fig. 1). The PR domain consists of a motif with ~20 amino acids, which can interact with Src homology‐3 domain (SH3 domain) involved in signal transduction and cytoskeleton regulation 25. SH3 is present in a large number of proteins involved in signal transduction, cell polarization and membrane‐cytoskeleton interactions 26. Thus, bioinformatics analysis shows that BCNP1 may be involved in various important protein‐protein interaction and cellular signalling pathways.

BCNP1 protein also contains a LZ domain located on the middle portion of the protein (Fig. 1B). The LZ domain was first described in 1988 27. They are involved DNA binding and regulate transcription. They are important for hetero‐ or homo‐ dimerization with related proteins containing LZ domains. They can also interact with other LZ domains containing proteins and regulate transcriptional activities. These findings indicate that BCNP1 may be involved in DNA binding and also dimerizes with other proteins to control transcriptional activity.

### Association of BCNP1 genetic alterations with the onset and progression of cancers

**Figure 2:**
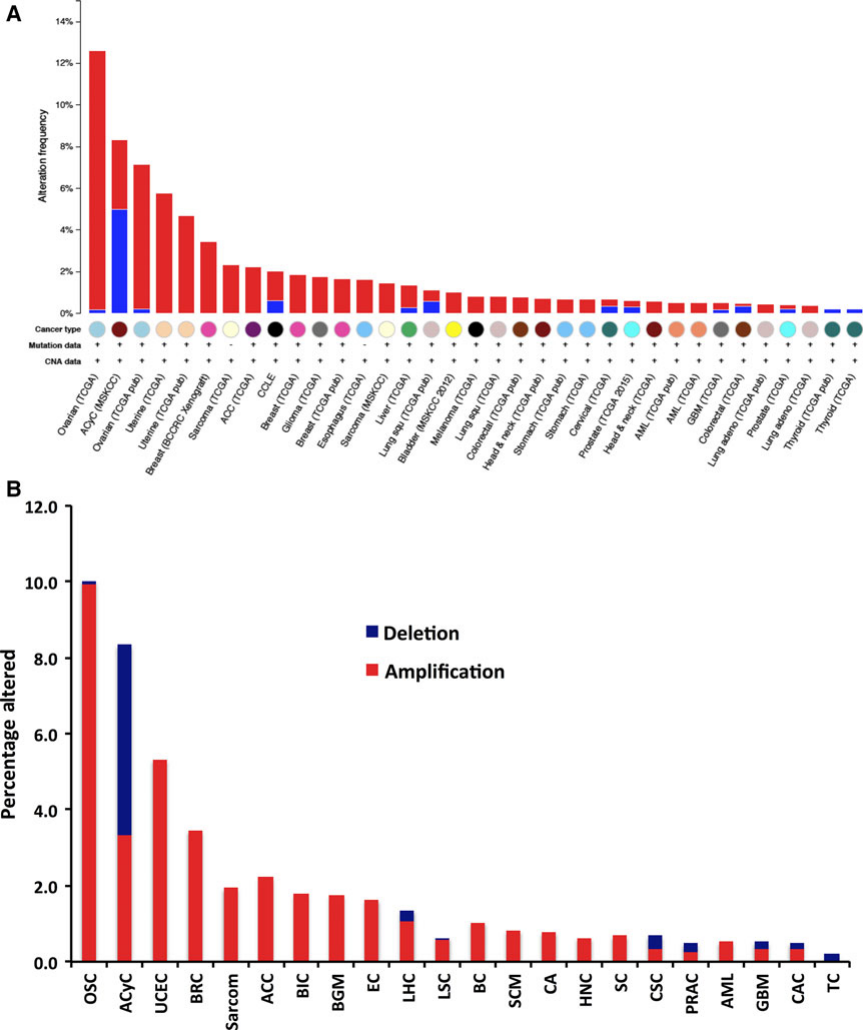
Copy‐number variations (CNV) in BCNP1 are frequent in human cancer. Cross‐cancer alteration summary for BCNP1 from 59 studies. The tumours analysed are: ovarian serous cyst adenocarcinoma (OSC), adenoid cystic carcinoma (ACyC), uterine corpus endometrial carcinoma (UCEC), breast cancer (BRC) (patient xenograft), sarcoma, adrenocortical carcinoma (ACC), cancer cell line encyclopedia (CCLE), breast invasive carcinoma (BIC), brain lower grade glioma (BGM), oesophageal carcinoma (EAC), liver hepatocellular carcinoma (LHC), lung squamous cell carcinoma (LSC), bladder cancer (BC), skin cutaneous melanoma (SCM), colorectal adenocarcinoma (CAC), head and neck squamous cell carcinoma (HNC), stomach adenocarcinoma (SC), cervical squamous cell carcinoma (CSC), prostate adenocarcinoma (PRAD), acute myeloid leukemia (AML), lung adenocarcinoma (LC), pancreatic adenocarcinoma (PAC) and thyroid carcinoma (TC). The data were obtained and analysed using cBioPortal. The amplifications and deletions were represented as red and blue bars, respectively. **(A)** CNV alterations of BCNP1 in the TCGA data sets for each cancer study. **(B)** Histogram illustrating the percentage of BCNP1 alterations for cancer studies combined according to cancer type.

To assess possible links between genetic alterations in BCNP1 and its outcome in transformation and cancer, we used TCGA data sets available from cBio‐Portal 19, 20. The TCGA cancer project is a well‐studied collection containing complete cancer patient study data, including clinical outcome data. Here, we compiled data from TCGA to see if functional activation or inactivation of BCNP1 might be an important event in most tumour types (Figs. 2 and 5).

Data for 59 cancer studies were collected to determine the percentage of cases wherein BCNP1 had copy‐number changes. Tumour types displaying a higher percentage of BCNP1 copy‐number changes include ovarian serous cystadenocarcinoma (OSC; 10%), adenoid cystic carcinoma (ACyC; 8.3%, 3.3% amplifications and 5% deletions), uterine corpus endometrial carcinoma (UCC; 5.3%) and breast cancer (BRC; 3.4%) (Fig. 2A and B).

**Figure 3:**
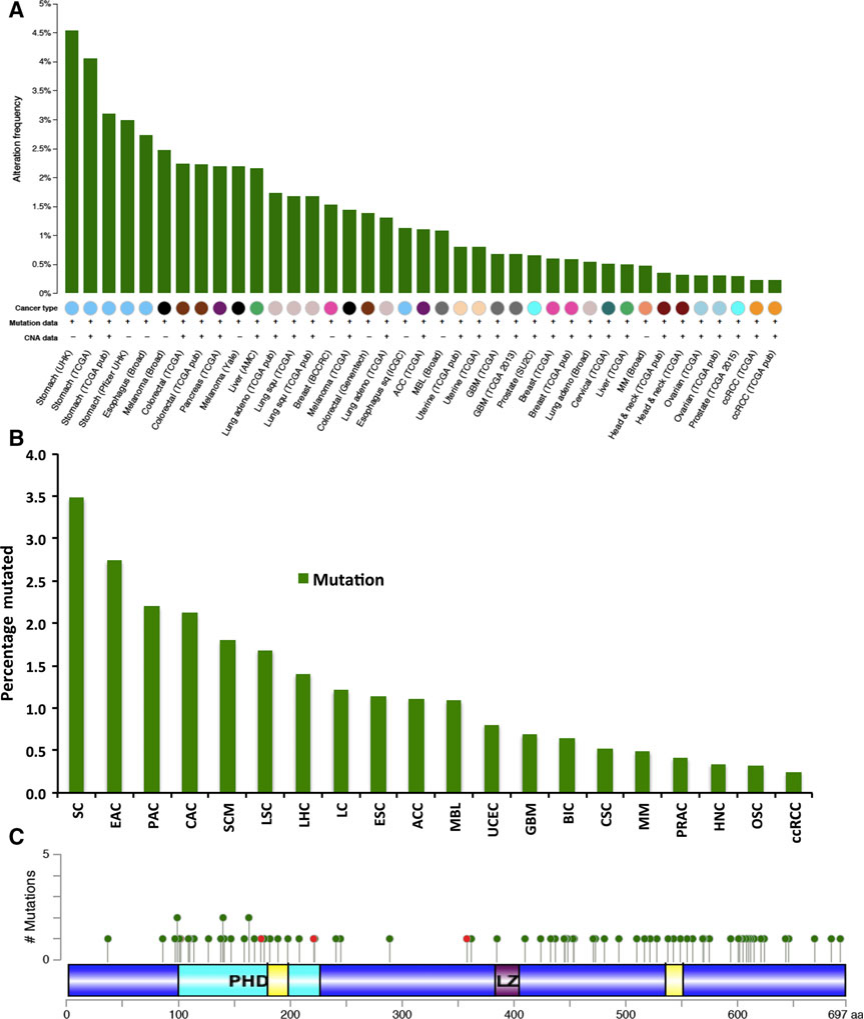
Mutation frequency of BCNP1 in human cancer. Cross‐cancer alteration summary for BCNP1 from 87 studies. The tumours analysed are: SC, EC, SCM, CAC, PRAD, LAC, LSC, BIC, ACC, medulloblastoma (MBL), UCEC, GBM, metastatic prostate cancer (PRAD), CSC, LHC, multiple myeloma (MM), HNSC, OSC, PRAD, kidney renal clear cell carcinoma (ccRCC). All the mutations were represented as green bar. The data were obtained and analysed by cBioPortal. **(A)** Mutations of BCNP1 in the TCGA data sets for each cancer study. **(B)** Histogram illustrating the percentage of BCNP1 mutations for cancer studies combined according to cancer type. **(C)** Somatic mutations found in BCNP1 gene by the TCGA studies. Circles represent the mutations: Nonsense, nonstop, frameshift deletion, frameshift insertion, and splice site (red) and missense mutations (green). The horizontal axis shows sites of mutations and vertical axis shows frequencies.

We also collected data to determine the percentage of cases wherein BCNP1 is mutated. The data shows the average of studies if there are of similar types. Tumour types having a higher percentage of BCNP1 mutations included stomach adenocarcinoma (SC; 3.5%), oesophageal carcinoma (EAC; 2.7%), pancreatic adenocarcinoma (PAC; 2.2%) and colorectal adenocarcinoma (CAC; 2.1%) (Fig. 3A and B). As shown in Figure 3C BCNP1 is also mutated mostly either at the PH domain or at the C‐terminal domain.

**Figure 4:**
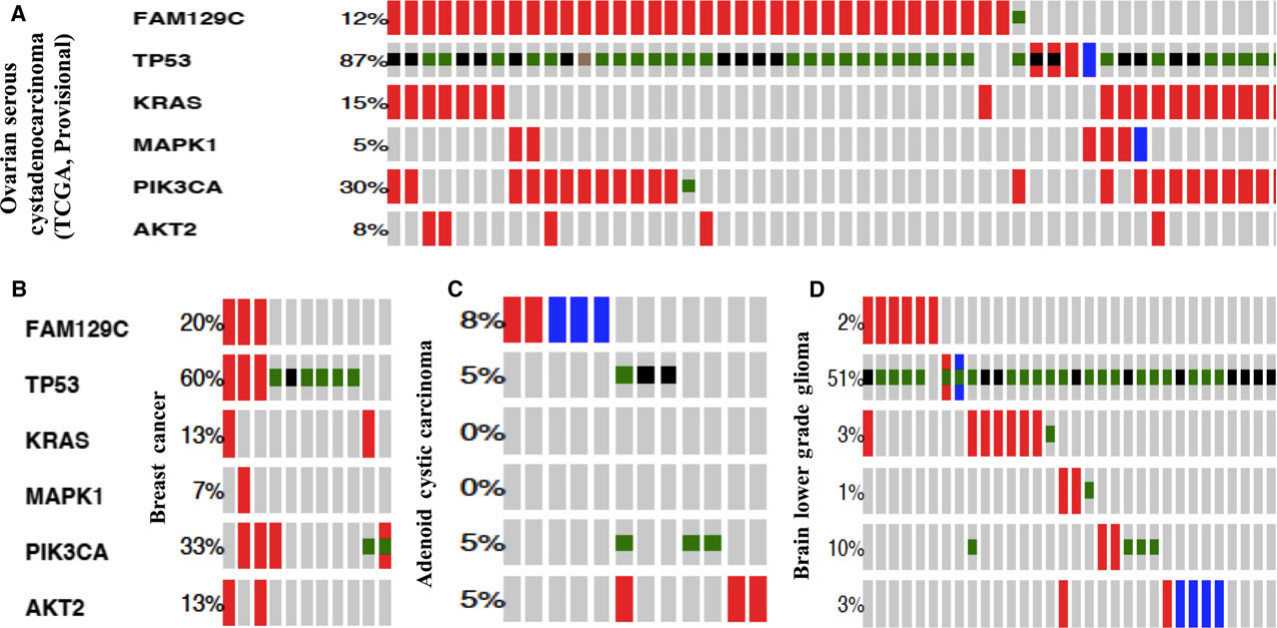
An OncoPrint showing the relationship of BCNP1 genetic alterations with TP53, KRAS, MAPK1 (ERK), PIK3CA (PI3K) and AKT2 mutational events in some of the cancer studies. Individual samples are represented as columns and individual genes are represented as rows. The alterations are represented as follows: amplification (red), deletion (blue), missense mutation (green), and truncating mutation (black). **(A)** BCNP1 alterations in OSC (TCGA, provisional; *n* = 311 samples) study have shown tendency towards co‐occurrence with those of TP53, KRAS, MAPK1, PIK3CA (PI3K), and AKT2 alteration events. **(B)** BCNP1 alterations in breast cancer (TCGA, provisional; *n* = 29 samples) study have shown tendency towards co‐occurrence with MAPK1 and AKT2 with significance of *P*‐0.015 and *P*‐0.042, respectively. **(C)** In ACyC (*n* = 60 samples) study, BCNP1 alterations were mutually exclusive with those of TP53, PIK3CA (PI3K) and AKT2 alterations. **(D)** In GBM (*n* = 286 samples) study, BCNP1 alterations were mutually exclusive with MAPK1, PIK3CA (PI3K) and AKT2 mutational events and have tendency towards co‐occurrence with those of TP53 and KRAS.

We examined the relationship between BCNP1 and other activating genetic alterations in the PI3K pathway. We also examined the role of ERK (extracellular signal regulated kinase) (MAPK1; mitogen‐activated protein kinase‐1) activation, KRAS amplification and TP53 amplification and mutation simultaneously. To generate an OncoPrint profile, we used data from various studies as follow: OSC (TCGA, provisional data), OSC (TCGA, Nature 2011), breast cancer (BC), adenoid cystic carcinoma (ACyC) and brain lower grade glioma (GBM). As shown in the Figure 4 A–D and Figure S1 OncoPrint, copy‐number changes and mutation events associated with BCNP1 have a tendency towards co‐occurrence with PI3KCA (PI3K) and AKT2. Alterations in BCNP1 are also generally complementary associated with other pathway events like KRAS and MAPK1 (ERK) amplification and TP53 mutations and amplifications. The mechanism of this co‐occurrence between BCNP1 and other pathway events remains unknown. Further biochemical studies are required to understand the role of BCNP1 in activation of various signalling pathways.

**Figure 5:**
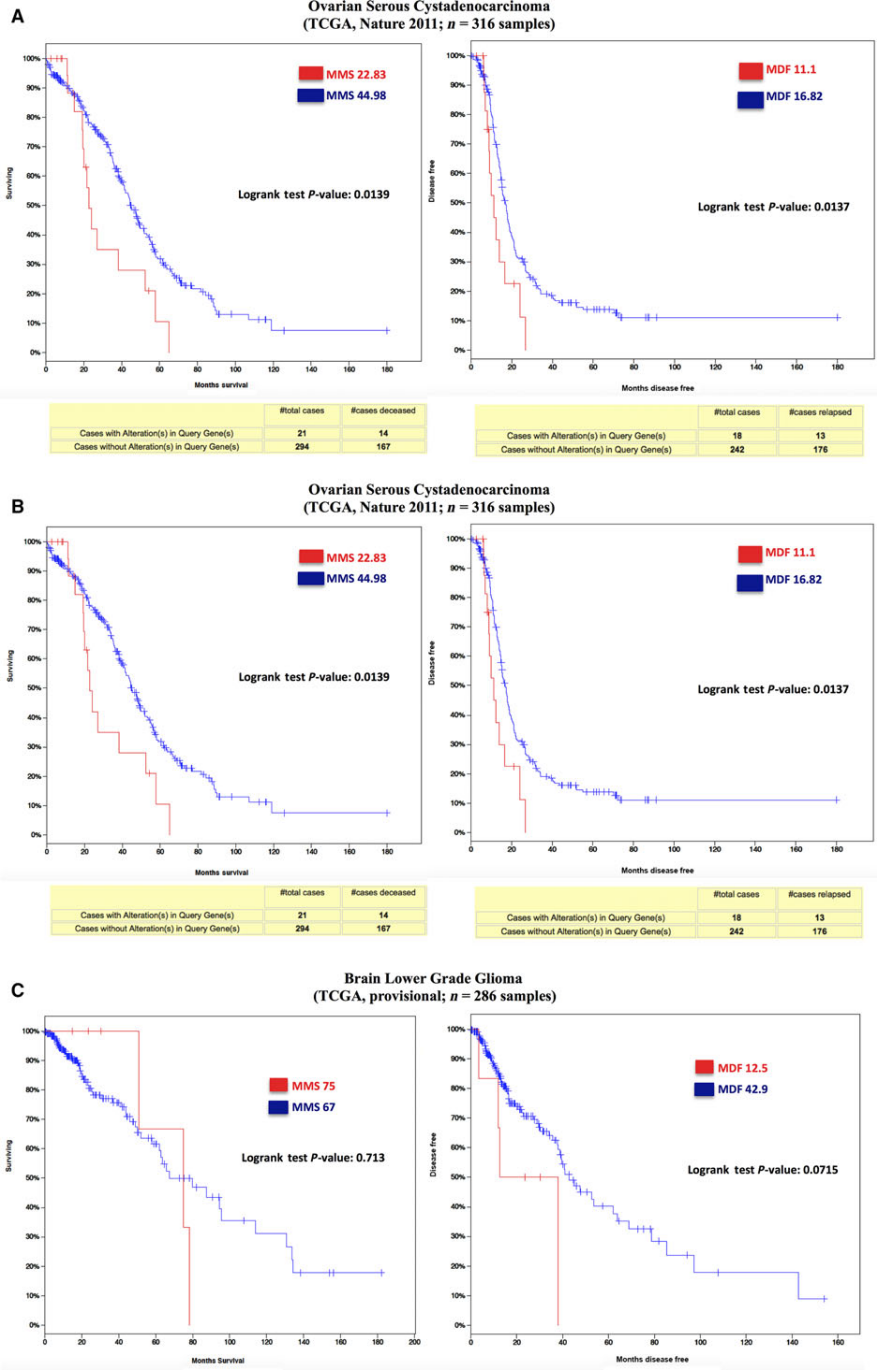
Survival analysis of altered BCNP1 in cancer cases. **(A)** The patients with the altered BCNP1 in OSC study (TCGA provisional) did not show significant differences in their overall survival (MMS) and disease‐free survival (MDF). **(B)** The patients with the altered BCNP1 in OSC (TCGA, Nature 2011) have a significantly (*P* = 0.0139) worse overall survival (MMS) and a significantly (*P* = 0.0137) worse disease free progression (MDF) compared to patients without any alterations in BCNP1. **(C)** The patients with the altered BCNP1 in GBM (TCGA, provisional) study did not show significant differences in their MMS and MDF. Although, the median disease free progression (MDF) was very poor with MDF of 12.5 months compared to MDF of 42.9 months in patients without BCNP1 alterations.

We also examined the combined impact of BCNP1 overexpression or mutation on clinical outcome in the TCGA data sets from OSC (TCGA provisional), OSC (TCGA, 28) and GBM (TCGA provisional) studies. When the TCGA data sets from OSC (TCGA, 28) study were divided into BCNP1 expression quintiles, the patients with the BCNP1 alterations had a significantly worse survival and poor disease free progression than the patients with no alterations with *P* = 0.0139 and *P* = 0.0137, respectively (Fig. 5B). In other studies, OSC (TCGA provisional) and GBM (TCGA provisional), there are not any significant differences in the patient survival and disease free progression (Fig. 5A and C). The disease free progression was poor in patients with BCNP1 alterations (12.5 months) compared to those without alterations (42.9 months) in the GBM (TCGA provisional) study (Fig. 5C). Taken together, this analysis demonstrates that BCNP1 alterations could be used as an indicator for the patient survival in some of the cancer cases.

### PI3K is upstream of BCNP1 and regulates its phosphorylation at serine residues

**Figure 6:**
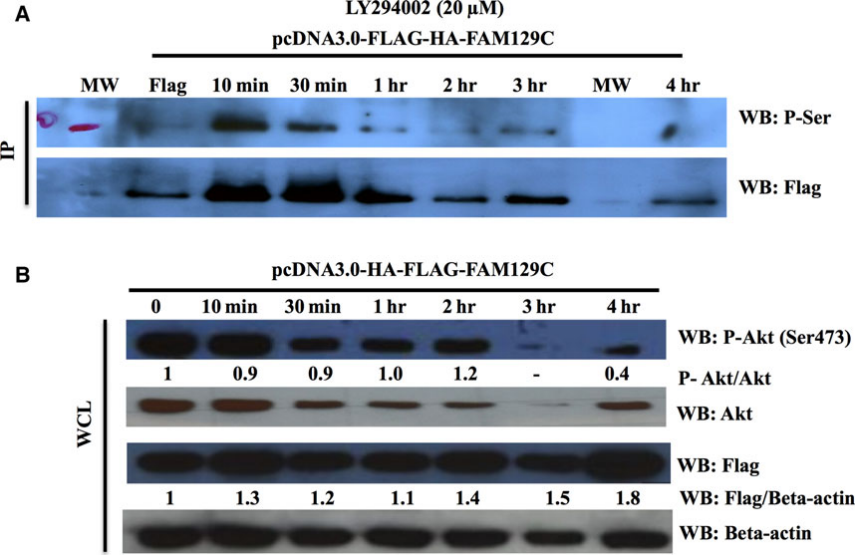
Effect of PI3K inhibition on BCNP1 phosphorylation at serine residues. (A) IP and (B) WB analyses were performed with the indicated antibodies. HEK293 cells were transfected with Flag‐HA‐BCNP1. After expression, cells were treated with indicated PI3K inhibitors and then incubated for the indicated period of time. Transiently expressing Flag‐HA‐BCNP1 HEK293 cells were treated with PI3K inhibitor LY294002 (20 μM) for 10 min., 30 min., 1, 2, 3, and 4 hrs. HA, haemagglutinin A; WCL, whole cell lysate; IP, immunoprecipitation; WB, western blot; MW, molecular weight marker.

Protein phosphorylation acts as an essential posttranslational modification regulating signalling pathways and various cellular functions like differentiation, cell movement, apoptosis, intercellular communication and cell survival 29. In particular, PI3K‐Akt pathways play an important role in growth and survival of cancer cells 5. Based on the co‐occurrence between PI3K and BCNP1 alteration, we examined the role of PI3K inhibition on BCNP1 phosphorylation. To determine whether BCNP1 phosphorylation is dependent on PI3K, we overexpressed HA‐Flag tagged BCNP1 into HEK293 cells and treated the cells with PI3K inhibitors. As shown in Figure 6 and Figure S2 transiently transfected HEK29 cells were treated with PI3K inhibitors LY294002 (20 μM) and Wortmannin (100 nM) 48 hrs post‐transfection. IP of Flag tagged BCNP1 with an anti‐FLAG antibody followed by WB with anti‐phosphoserine revealed that PI3K inhibition reduced phosphorylation of BCNP1 at serine residues in time‐dependent manner. We also confirmed PI3K inhibition by blotting against anti‐phosphorylated Akt and in WCL. We also used anti‐FLAG antibody to determine the level of overexpression in WCL. These results indicate that PI3K acts upstream of BCNP1 and regulates BCNP1 phosphorylation at serine residues.

### BCNP1 phosphorylation at serine residues is dependent on p38 MAPK activation

**Figure 7:**
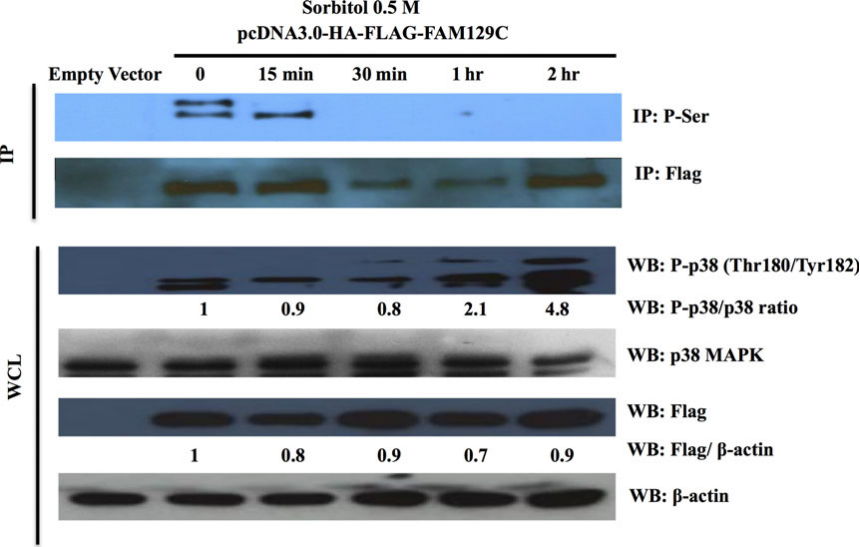
p38 MAPK‐mediated activation decreases BCNP1 phosphorylation at serine residues. HEK293 cells were transiently transfected, treated with 0.5 M sorbitol as indicated at different time points, and analysed by IP and WB. Osmotic shock leads to a profound downregulation of BCNP1 serine‐phosphorylation. The phosphorylated BCNP1/BCNP1 ratio is indicated. Ten percentage of the IP input was analysed by WB (upper panel). Osmotic shock also leads to a slight reduction in BCNP1 protein levels. Flag tagged proteins were detected by WB (lower panel).

Next, we investigated whether p38 MAPK activation leads to change in BCNP1 phosphorylation at serine residues. Treatment of transiently transfected HEK293 cells with 0.5 M sorbitol, a well‐established osmotic shock inducer and selective p38 MAPK activator, led to a ~10–30% reduction of BCNP1 protein level (Fig. 7). As expected, p38 MAPK activation occurred at early time points after treatment. Furthermore, we found that sorbitol induced a striking reduction in serine phosphorylated BCNP1 that was maximal after ~30 min. of treatment. Thus, we concluded that sorbitol‐induced BCNP1 serine‐phosphorylation is dependent on p38 MAPK activity.

### Halflife and proteasome‐mediated degradation of BCNP1

**Figure 8:**
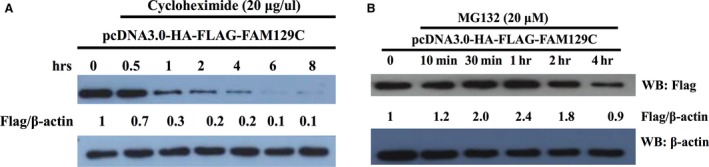
BCNP1 is degraded in a proteasome‐dependent manner. **(A)** BCNP1 undergoes degradation in transiently transfected HEK293 cells. Overexpressed Flag‐BCNP1 and actin proteins were detected by WB in cycloheximide (20 μg/ml) treated cells. **(B)** Transiently transfected HEK293 cells were treated with MG132 (20 μM) as indicated. Proteasome inhibition leads to BCNP1 up‐regulation. Overexpressed Flag‐BCNP1 and actin were detected by WB.

We also determined whether BCNP1 is degraded in transiently transfected HEK293 cells using the protein synthesis inhibitor cycloheximide at 20 μg/μl. Treatment led to decrease in BCNP1 protein levels with a half‐life of ~45 min. in transiently transfected HEK293 cells (Fig. 8A).

Next, treatment of transiently transfected HEK293 cells with proteasome inhibitors such as MG132 at 20 μM led to increased BCNP1 level doubled after 30 min. (Fig. 8B). We concluded that BCNP1 might undergo degradation in proteasome‐mediated fashion.

## Conclusion

**Figure 9:**
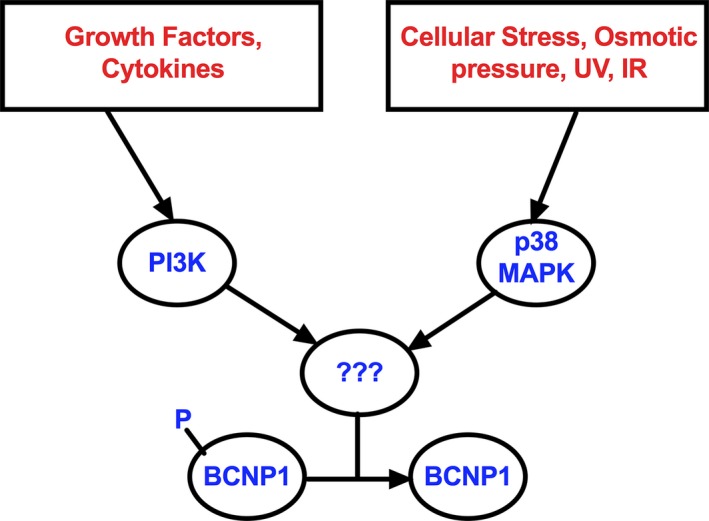
Molecular mechanisms controlling BCNP1 regulation. During the cellular response to stress controls BCNP1 protein levels may be through p38 MAPK. BCNP1 serine‐phosphorylation is downstream of PI3K and p38 MAPK signals.

BCNP1 is a not very well studied protein with unknown function(s). BCNP1 is more highly expressed in cancer samples than normal tissues 1. However, very little is known regarding the mechanisms that control BCNP1 protein levels post‐transcriptionally, which is of critical relevance since BCNP1 is frequently amplified or mutated in prevalent human malignancies. Our bioinformatics mining suggests that BCNP1 has a PH domain, two PR rich regions and LZ domain potentially involved in protein‐protein interactions, dimerization and cell signalling. Functionally, our biochemical data shows that BCNP1 is regulated by the PI3K pathway and phosphorylated at serine residues as illustrated in model (Fig. 9). Therefore, the PH domain and the two PR rich regions could be involved in protein‐protein interactions that are part of PI3K pathway, in particular transient protein‐protein interactions.

The p38 MAPK pathway plays an essential role in regulating inflammation, cell differentiation, cell growth and apoptosis. It is strongly activated in response to stress stimuli such as osmotic shock, UV and ionizing radiation, and inflammatory cytokines. It might have different roles depending on cellular context and can play an important role in cancer. Our work defines a functional connection between p38 MAPK and BCNP1 (Fig. 9). Using Kinase Phos 2.0 prediction tool 30, we postulate that the BCNP1 can be phosphorylated at serine residues 37 and 76 using p38 MAPK. The serine 37 position in BCNP1 was also found to be mutated (S37L) in the some of the colon cancer patient samples as searched using TCGA data sets.

Genomic efforts to categorize human tumour types have provided further understanding into the molecular contribution of heretofore understudied gene products of BCNP1. Our findings revealed that BCNP1 is overexpressed in many types of human cancer cells. Undoubtedly, cell type‐specificity and even type of genetic alterations in tumours are critical determinants of the cell signalling pathways mediated by BCNP1. Given the relative frequency of BCNP1 alterations in many cancer types warrant further investigation of its function providing answers to question when BCNP1 is amplified, deleted or mutated. Our recent observation that BCNP1 (FAM129C) was the most highly up‐regulated gene when non‐growing low density Ph+ ALL3 cells were stimulated to proliferate by cell‐free supernate from the same cells growing rapidly at a high cell density, and Yuki's report that overexpression of ZNF777 inhibited proliferation of cells at low density by down‐regulating FAM129A, suggests that BCNP1 probably has a very important role in cell‐cell communication and corresponding intracellular signalling pathways 16, 17, 18.

## Conflicts of interest

We declare that we have no conflict of interest.

## Supporting information


**Fig. S1.** An OncoPrint showing the relationship of BCNP1 genetic alterations with TP53, KRAS, MAPK1 (ERK), PIK3CA (PI3K) and AKT2 mutational events.
**Fig. S2.** Effect of PI3K inhibition on BCNP1 phosphorylation at serine residues.
