# Supplementary material for: The possible roles of B‐cell novel protein‐1 (BCNP1) in cellular signalling pathways and in cancer
Source: J Cell Mol Med. 2016 Sep 29;21(3):456–66. doi: 10.1111/jcmm.12989 (PMC5323820; doi:10.1111/jcmm.12989)
Supplement: Supplementary file 1 — Fig. S1. An OncoPrint showing the relationship of BCNP1 genetic alterations with TP53, KRAS, MAPK1 (ERK), PIK3CA (PI3K) and AKT2 mutational events. Fig. S2. Effect of PI3K inhibition on BCNP1 phosphorylation at serine residues. [file JCMM-21-456-s001.docx]

**The Possible Roles of B-Cell Novel Protein-1 (BCNP1) in Cellular Signaling Pathways and in Cancer**

Sapan J Patel^1,2^, Gaurang L Trivedi^3^, Costel C. Darie^2^, Bayard D. Clarkson^1¶^

^1^Memorial Sloan Kettering Cancer Center

Molecular Pharmacology and Chemistry Program

1275 York Ave, Box 96,

New York, NY 10021

^2^Clarkson University

Biochemistry and Proteomics Group

Department of Chemistry and Biomolecular Science

Clarkson University

8 Clarkson Avenue

Postdam, NY 13699-5810

^3^Cold Spring Harbor Laboratory

1 Bungtown Road

Cold Spring Harbor, NY 11724


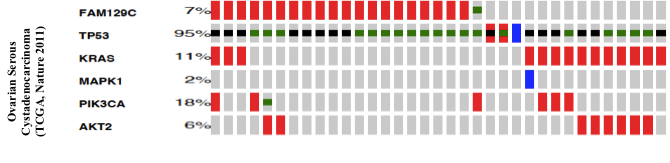


**Supplementary Figure 1:**

An OncoPrint showing the relationship of BCNP1 genetic alterations with TP53, KRAS, MAPK1 (ERK), PIK3CA (PI3K) and AKT2 mutational events. Individual samples are represented as columns and individual genes are represented as rows. The alterations are represented as follow: amplification (red), deletion (blue), missense mutation (green), and truncating mutation (black).

BCNP1 alterations in OSC (TCGA, Nature 2011; n=316 samples) study have shown tendency towards co-occurrence with those of TP53, KRAS, PIK3CA (PI3K) and AKT2 alterations and is mutually exclusive with those of MAPK1 alteration events.


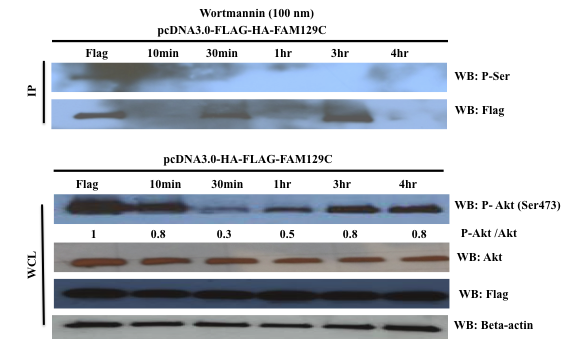


**Supplementary Figure 2:**

Effect of PI3K inhibition on BCNP1 phosphorylation at serine residues. IP and WB analyses were performed with the indicated antibodies. HEK293 cells were transfected with Flag-HA-BCNP1. Transiently expressing Flag-HA-BCNP1 HEK293 cells were treated with PI3K inhibitor wortmannin (100 nM) for 10 min, 30 min, 1, 3, and 4 hr. HA, haemagglutinin A; WCL, whole cell lysate; IP, immunoprecipitation; WB, western blot; MW, molecular weight marker.
